# Resource partitioning of a Mexican clam in species-poor Baltic Sea sediments indicates the existence of a vacant trophic niche

**DOI:** 10.1038/s41598-024-62832-3

**Published:** 2024-05-31

**Authors:** Agnes M. L. Karlson, Nils Kautsky, Matilda Granberg, Andrius Garbaras, Hwanmi Lim, Camilla Liénart

**Affiliations:** 1 Department of Ecology, Environment and Plant Sciences, Stockholm University, Stockholm, Sweden; 2 Stockholm University Baltic Sea Centre, Stockholm, Sweden; 3 Center for Physical Sciences and Technology, Vilnius, Lithuania; 4 Lipidor AB, Svärdvägen 13, 182 33 Danderyd, Sweden; 5 Université de Bordeaux, CNRS, Bordeaux INP, EPOC, UMR 5805, 33120 Arcachon, France

**Keywords:** Food partitioning, Alien species, Benthic-pelagic coupling, Stable isotopes, Fatty acids, Benthic bivalves, Ecology, Environmental sciences, Natural hazards

## Abstract

Invasive species are often generalists that can take advantage of formerly unexploited resources. The existence of such vacant niches is more likely in species-poor systems like the Baltic Sea. The suspension feeding wedge clam, *Rangia cuneata*, native to estuarine environments in the Gulf of Mexico, was sighted for the first time in the southeastern Baltic in 2010 and a few years later in the northern Baltic along the Swedish coast. To explore possible competition for food resources between *R. cuneata* and the three native clams inhabiting Baltic shallow soft bottoms, stable isotope and fatty acid analyses were conducted. There was no overlap between *R. cuneata* and any of the native species in either stable isotope or fatty acid niches. This suggests efficient partitioning of resources; multivariate analyses indicate that separation was driven mainly by δ^13^C and by fatty acids reflecting diatoms and cyanobacteria, respectively (e.g. 16:1ω7 and 18:3ω3). *R. cuneata* reflected seasonal variation in phytoplankton more than other clams reflecting higher trophic plasticity. In conclusion, the addition of *R. cuneata* to the Baltic shallow soft bottoms suggests the existence of a vacant trophic niche in these sediment habitats, however the long-term effects on other species and nutrient cycling requires further studies focusing on the population dynamics of *R. cuneata* and its impact on the Baltic Sea ecosystem.

## Introduction

Invasive species are considered a major threat to biodiversity and may alter ecosystem functioning^1–4^. Co-existence of species is, according to classic ecological theory, built on niche differentiation^5^. One example of detrimental impact of non-indigenous species is when they share a trophic niche with the native species but are competitively superior, for instance, by having higher feeding rates or superior food conversion efficiency resulting in higher production^6,7^, or from interference competition for space^8^. Not all non-indigenous species are however categorized as invasive. The existence of vacant niches can allow for a non-indigenous species to enter a new habitat without competing with the native species^9,10^. Non-indigenous species that are successful are often generalists that can take advantage of formerly unexploited resources, thereby occupying a distinct niche space relative to the native community^7,11^ and potentially making resource utilization more efficient^9,10^.

To investigate the trophic ecology of consumers, a key aspect of the ecological niche, the analysis of carbon and nitrogen stable isotope composition is a commonly used technique. The carbon isotope ratio (δ^13^C: ^13^C/^12^C) of a primary consumer will reflect the carbon signal of the ultimate primary producer, while the nitrogen isotope ratio (δ^15^N: ^15^N/^14^N) is indicative of the trophic position of the organism^12^. The δ^13^C and δ^15^N values can be plotted in a two-dimensional isotopic space for several individuals of a given species, and the area which the discrete values of individuals occupy represents the species’ isotopic niche (proxy for the trophic niche^13–15^). Species with a high variation in diet among individuals will have a larger isotopic niche than species with a more uniform/narrow diet^10^. If two species show a large overlap of their isotopic niche, they might share similar diets^10^. However, the δ^13^C and δ^15^N values among different potential food sources can be similar, making diet interpretation difficult. Furthermore, variation in physiological status among individuals will also influence the isotope niche size through metabolic fractionation, further complicating the accurate representation of the trophic niche^16,17^. A complement to isotope analyses is provided by fatty acid analyses^18–21^. Although more labor intensive and costly, fatty acids can function as biomarkers of certain groups of primary producers^22^, which have similar isotope composition. Combining the two approaches (stable isotope and fatty acid analyses) provide a robust estimate of the trophic niche of consumers.

Brackish systems with low species richness seem to be more vulnerable to non-indigenous species invasions and the Baltic Sea has been described as a sea of aliens^23,24^ with the existence of vacant niches^9,10^. The non-indigenous wedge clam, *Rangia cuneata* is a soft bottom suspension feeding bivalve native to sub-tropical estuarine environments in the Gulf of Mexico, but has spread in ballast water to European brackish and freshwater regions and was found for the first time in the south-eastern Baltic in 2010^25–27^. It seems to occupy shallow silty sediments in the Baltic, and in these low saline habitats *R. cuneata* often, but not always, represents the only obligate suspension feeding bivalve^28^. There are yet no comprehensive studies of its dietary habits and potential for resource competition with native species. Recent research on this species in the Baltic include the metabolic responses to the low temperature and salinity^29^, health status^30^, mercury uptake^31^ and population genetic structure^32^. Sightings of *R. cuneata* along the Swedish coast occurred first in 2016 and has since been observed in several regions in southern Sweden^33^. There are only three abundant macrofaunal bivalve species that currently inhabit the same habitat (shallow soft sediments) that *R. cuneata* has moved into, the suspension feeders *Cerastoderma glaucum* and *Mya arenaria* and the facultative suspension deposit feeder *Macoma balthica*. Since *R. cuneata* has a habitat overlap with these three native species in the Baltic, it is important to know if they might also overlap in their diet. The aim of this study is to test for the first time the possible dietary overlap using isotope and fatty acid biomarkers between the non-indigenous *R. cuneata* and the three indigenous soft bottom clams, in the Baltic Sea at two sites, and whether the overlap changes over the productive season. We expect these generalist suspension-feeders to show significant dietary overlap and the native facultative suspension-deposit-feeding species to have the broadest niche size.

## Methods

### Field collection and size measurements of clams

**Figure 1:**
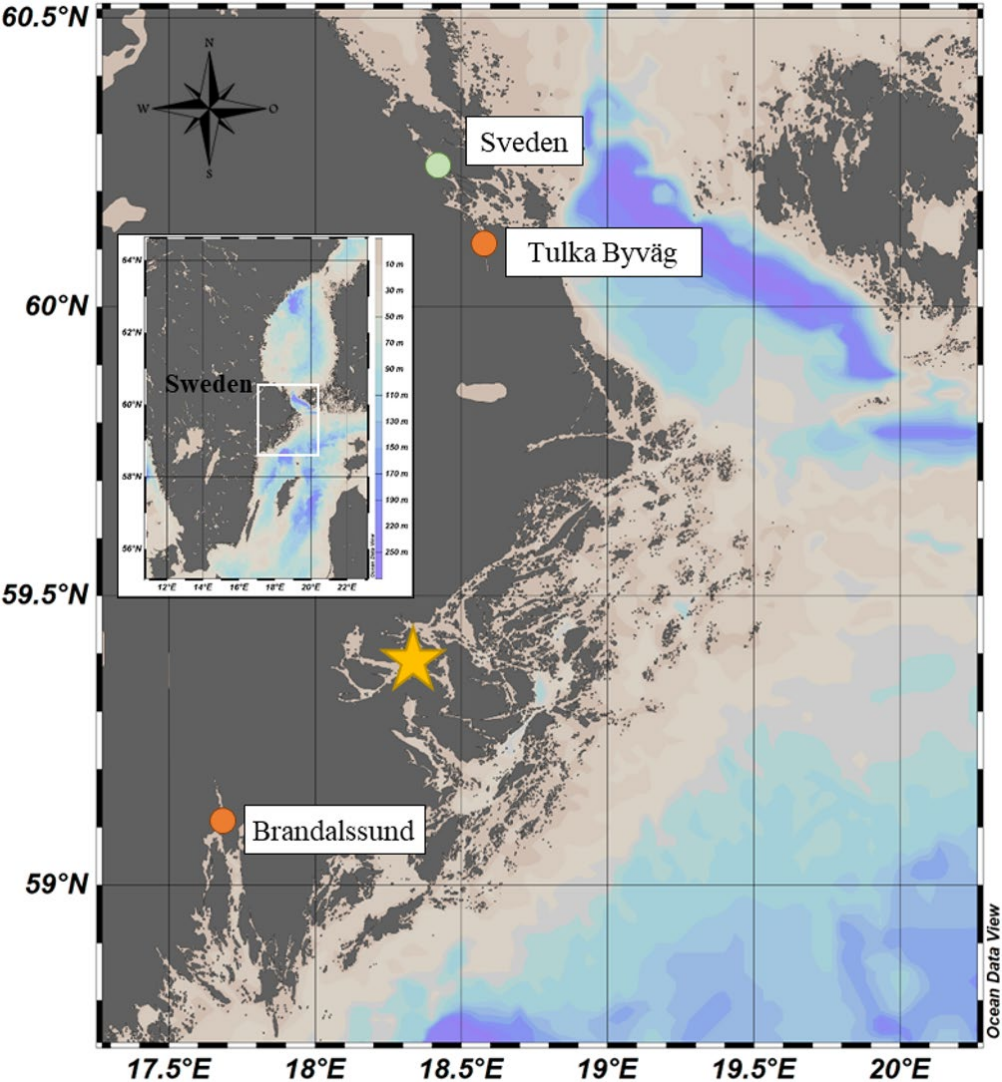
Map of the three sampling locations of the native (*C. glaucum, M. balthica, M. arenaria*) and non-native (*R. cuneata*) clams. Sveden (one timepoint; only *R. cuneata* found), Tulka Byväg (one timepoint, *R. cuneata* and *M. balthica* found), and Brandalssund (three timepoints; all species found). Stations in orange were analyzed for stable isotopes, fatty acids, condition index and age structure, the station in green was only sampled for age distribution determination. The yellow star represents Stockholm city. The map was generated with Ocean Data View (Schlitzer 2021, https://odv.awi.de/, version 5.5.1).

We sampled three locations along the Swedish east coast (Fig. 1) which represent the northernmost distribution range of *R. cuneata* in the Baltic. Brandalssund is located in the southern Stockholm archipelago (sandy bottom), and Tulka Byväg and Sveden are located in the northern Stockholm archipelago (muddy bottoms). Brandalssund is where the highest abundances of individuals of all species were found and was sampled at three timepoints in 2021, i.e. in spring (April), in early summer (end of June/early July) and in late summer (August/September). Samples collected at Sveden and Tulka Byväg were collected in late summer only (September); at Sveden only *R. cuneata* individuals were found and at Tulka the only other species found was *M. balthica*.

The clams were collected between 0.4 and 1 m depth using a shovel and a sieve with a 2 mm mesh size at Brandalssund and with 10 mm mesh size at Tulka Byväg and Sveden. The mesh size differed between sites because of the sediment grain size differences. Adult bivalves are larger than 10 mm and thus retrieved with both sieves. During collection, the samples were stored in a cooling box until being placed in a − 20 °C freezer the same day (− 80 °C for clams intended for fatty acid analyses, see below). *Rangia cuneata* individuals sampled in late summer from all three sites were measured for length and width using a caliper, and age was determined by counting the darker growth rings on the shell of the clams. To measure condition index, shells and soft tissue (full body) of the clams were weighed after drying at 60 °C (soft tissues were kept for later isotope analyses); the clam content to shell weight (dry soft tissue:shell ratio) was used as a proxy for clams’ condition^34^. The number of growth rings on the clam shells were counted to compare age structure among sites and to gain perspective of potential differences in establishment time of each population.

### Stable isotope analyses

We analyzed dried clam material (muscle tissue only) of 14–20 individuals per species and station and timepoint, except for *Mya arenaria*, which was only found at one site and timepoint in lower numbers n = 4 only. Samples were weighed in tin caps and analyzed for carbon and nitrogen elemental and stable isotope content at the Center for Physical Science and Technology (Vilnius, Lithuania), using an Elemental Analyzer (Flash EA1112 Series, Thermo Finnigan) connected to an Isotope Ratio Mass Spectrometer (DeltaV Advantage, Thermo Finnigan). Ratios of C and N stable isotopes are expressed as per mille deviation (‰) from international standard (Vienna Pee Dee belemnite for δ^13^C and atmospheric N_2_ for δ^15^N) using Eq. (1):

1 $$\updelta ^{{{13}}} {\text{C}}_{{{\text{sample}}}} \;{\text{or}}\;\updelta ^{{{15}}} {\text{N}}_{{{\text{sample}}}} = [({\text{R}}_{{{\text{sample}}}} /{\text{R}}_{{{\text{standard}}}} ) - {1}] \times {1}000$$

where R is the ratio of heavy/light isotopes, ^13^C/^12^C or ^15^N/^14^N. External (Caffeine IAEA600) and internal standards were analyzed as references every 10 samples, within each batch of samples. Analytical error were < 0.15‰ and < 0.20‰ for δ^13^C and δ^15^N, respectively.

Isotope niche analyses (standard ellipse area corrected for small sample size, SEAc, and Bayesian standard ellipse area, SEA_B_,^13^) were calculated using the R package ‘SIBER’ (version 2.1.5.). For all species, except *M. arenaria,* sample size was sufficient for robust niche analyses according to^13^ (niche results of *M. arenaria* are shown but should hence be interpreted with caution). The overlap in niche size among species within a site and sampling timepoint was calculated with the maximum likelihood standard ellipse area (given as the percentage of total area, i.e. considering areas of both ellipses being compared). Bayesian credible intervals were calculated to compare niche sizes. See “Statistics” section for statistical analyses.

### Fatty acid analyses

Fatty acid analyses require more material than isotope analyses and apart from *R. cuneata*, only *M. balthica* were found in enough numbers at Brandalssund at all seasons for this purpose. However, in late summer *C. glaucum* was also included since enough individuals were found this timepoint. The clam soft tissues were removed from their shell, and five clams from the same species and timepoint were pooled for fatty acid analyses. The samples were stored at − 80 °C, thereafter freeze dried and weighed to allow quantitative estimates of fatty acid content. Total lipids from the samples were first extracted, followed by fatty acids derivatization (i.e., methylation of FAs, resulting in FA methyl ester; see^35^ for protocol). The samples were then analyzed using gas chromatograph-flame ionization detection (GC/FID) with YL 6100 GC (YL Instruments Co., Ltd) using an Agilent DB-WAX column (30 m length, 0.32 mm internal diameter, 0.25 μm film thickness) with a slight modification in the temperature program: the initial temperature was 40 °C for 3 min and increased to 150 °C (50 °C/min), then to 205 °C (5 °C/min) and kept for 15 min. Then, temperature was increased to 250 °C (50 °C/min) and kept for 5 min. To quantify fatty acids, the 13-carbon chain long fatty acid (C13:0) was used as an internal standard. The results were given in fatty acid methyl ester per gram sample for each fatty acid, which allows for comparisons between species and timepoints, and was then recalculated into % of the total fatty acid per sample.

### Statistical analyses

Statistical analyses were performed in R 4.0.3 or Statistica 13.3 (Statsoft, Tibco). After inspection of normality and homoscedasticity of the residuals (Shapiro–Wilk and Levene tests, respectively) comparisons between species, timepoints, and sites, were performed with non-parametric Kruskal–Wallis or Mann–Whitney Wilcoxon tests (reported in the results as KW and MW, respectively) as the criteria for parametric tests were not met. The comparison of *R. cuneata* age distribution between the three sites (Tulka Byväg, Sveden and Brandalssund) was carried out using a Kruskal–Wallis rank-sum test. The condition index (soft tissue:shell ratio) of the clams (each species) was compared between the three timepoints in Brandalssund using Kruskal–Wallis rank-sum test (one test per species). To compare the different δ^13^C and δ^15^N values between the different species and seasons at site Brandalssund, and between species at site Tulka Byväg, a Kruskal–Wallis test was used. To analyze possible differences in the proportion of some of the fatty acids of relevance (e.g., biomarkers of diatoms and cyanobacteria) among species, Kruskal–Wallis test was used. Finally, to compare fatty acids profiles (all fatty acids) between the different species and timepoint, a non-metric multidimensional scaling ordination plot (nMDS, R package ‘vegan’, version 2.5.6.) was performed based on the Bray–Curtis dissimilarity matrix. Differences between species, timepoint and the interaction of both were investigated with a permutational multivariate ANOVA (PERMANOVA, 9999 permutations, on distance matrix previously tested for homoscedasticity, on the nMDS plot ellipses set to 40% of the data since each datapoint consisted of a composite sample of five individuals). A similarity percentages analysis (SIMPER, 9999 permutations) was performed to identify which FAs were responsible for 80% of the average dissimilarity between the most significant factor (species). The fatty acids that together explained 80% of the variation between species were investigated to determine possible dietary differences.

## Results

### Site specific age distribution and temporal change in body condition for *R. cuneata*

The density of *R. cuneata* at the investigated sites were 20–40 individuals per m^2^, and about the same for *M. balthica* (the study was not designed to quantitatively determine density since that would have required benthic cores with a large diameter and snorkeling). The other two species were found in much lower numbers (< 1 ind. per m^2^) and only at the site Brandalssund. The median age for *R. cuneata* collected at Brandalssund was 5 years, for Tulka Byväg 4 years and for Sveden 2 years (Fig. S1). There was no difference in body condition for *R. cuneata* (soft tissue:shell weight, Fig. S2) collected in Brandalssund between the three different timepoints (KW, H_(2, 60)_ = 4.64, p = 0.09) or for *C. glaucum* (not found in spring, MW, Z_(1, 34)_ = 1.38, p = 0.16). *M. balthica* had however a lower body condition in late summer (compared spring and early summer: KW, H_(2, 60)_ = 17.87, p < 0.001).

### Species specific difference in isotope composition across sites

**Figure 2:**
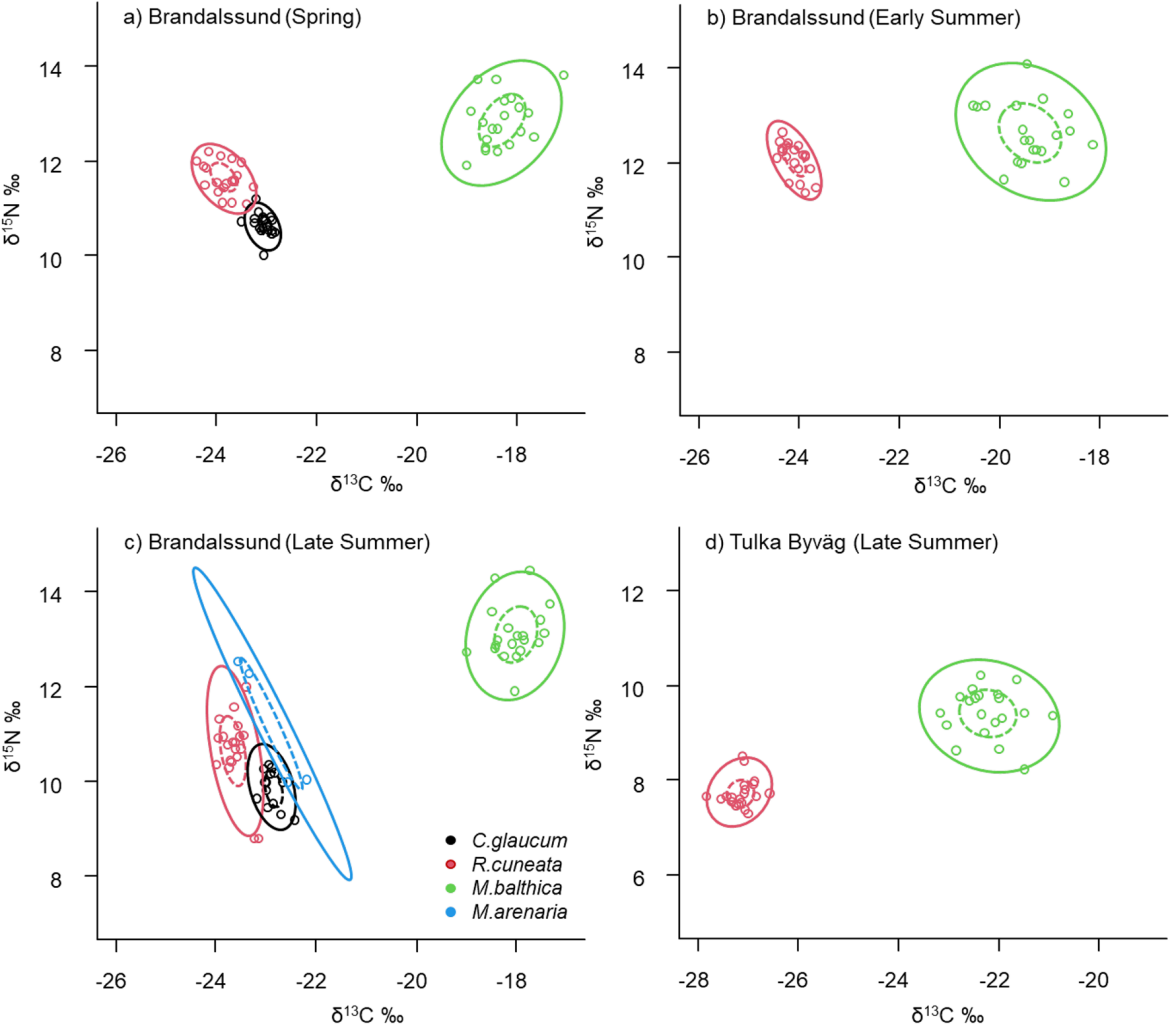
Isotope niches (SEAc: dash lines represent the core trophic niche i.e. 40% of the data, solid line represents 95% of the data) for the native (*C. glaucum* (black)*, M. balthica* (green)*, M. arenaria* (blue)) and non-native (*R. cuneata* (red)) clams sampled in (**a**) spring, (**b**) early summer and (**c**) late summer at Brandalssund, and (**d**) in late summer at Tulka Byväg.

Isotope niche analyses revealed very low overlap among *R. cuneata* and the native clams at the two studied locations (Fig. 2c, d, 0% when assessed with the 40% core isotope niche^13^ or when assessed with 95% ellipses,7.7% with *C .glaucum*, 4.6%, with *M. arenaria* and 0% with *M. balthica*). At Brandalssund, which was sampled throughout the season, the niche separation was stable over time, i.e. no overlap (< 1%) recorded in spring or early summer either (Fig. 2a, b). Overlapping ellipses occurred between native clams *C. glaucum* and *M. arenaria* in Brandalssund in late summer (Fig. 2a, 6.2%).

**Figure 3:**
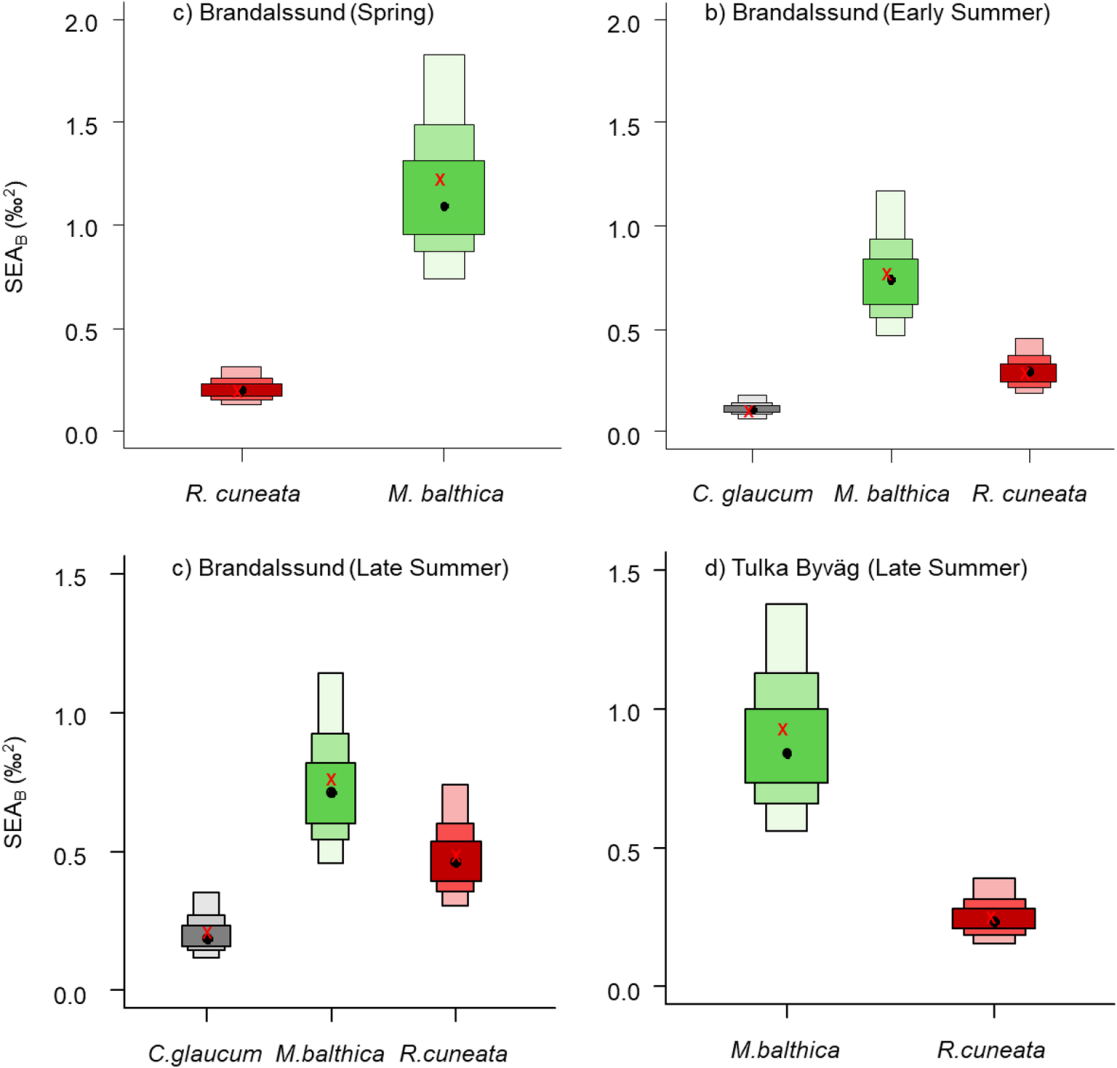
Bayesian estimates for the Standard Ellipse Area (SEA_B_) for the native (*C. glaucum* (black)*, M. balthica* (green)) and non-native (*R. cuneata* (red)) clams sampled in (**a**) spring, (**b**) early summer and (**c**) late summer at Brandalssund, and (**d**) in late summer at Tulka Byväg. The boxes represent the credibility intervals of 50%, 90% and 95%. Black dots represent the mean, red crosses represent the maximum likelihood estimated corrected standard ellipse area (SEAc). *M. arenaria* was not included in the density plots since there were too few individuals (the uncertainty estimates would be large) but raw data and the ellipses are shown for visual purposes in the bi-plots in Fig. 2.

The negligible niche overlap between *R. cuneata* and the other species were mainly driven by its more depleted ^13^C values compared to the other species at both sites (Fig. 2). At Brandalssund, *M. balthica* always had a higher δ^13^C values (Fig. 2) than *R. cuneata* and the other clams (KW, H_(2, 157)_ = 135.40, p < 0.001. *M. balthica* had also higher δ^15^N values than the other three species (KW, H_(2, 157)_ = 112.59, p < 0.001). The higher isotope values for *M. balthica* relative to *R. cuneata* were found also at site Tulka Byväg (δ^13^C: MW, Z_(1, 40)_ = 5.40, p < 0.001; δ^15^N: MW, Z_(1, 40)_ = 5.34, p < 0.01). *R. cuneata* had higher δ^15^N and lower δ^13^C values than *C. glaucum* in spring, less pronounced so in late summer (Fig. 2). Regarding niche size (Fig. 3), there was no clear difference between *R. cuneata* and *M. balthica* at Brandalssund in late summer, and *C. glaucum* had a smaller niche compared to the other species. *M. balthica* always showed a larger trophic niche than the other species. At Tulka Byväg, *R. cuneata* had a smaller niche than *M. balthica* and this pattern was also found in spring and early summer at Brandalssund (Fig. 3).

### Temporal change in isotope composition for clams

**Figure 4:**
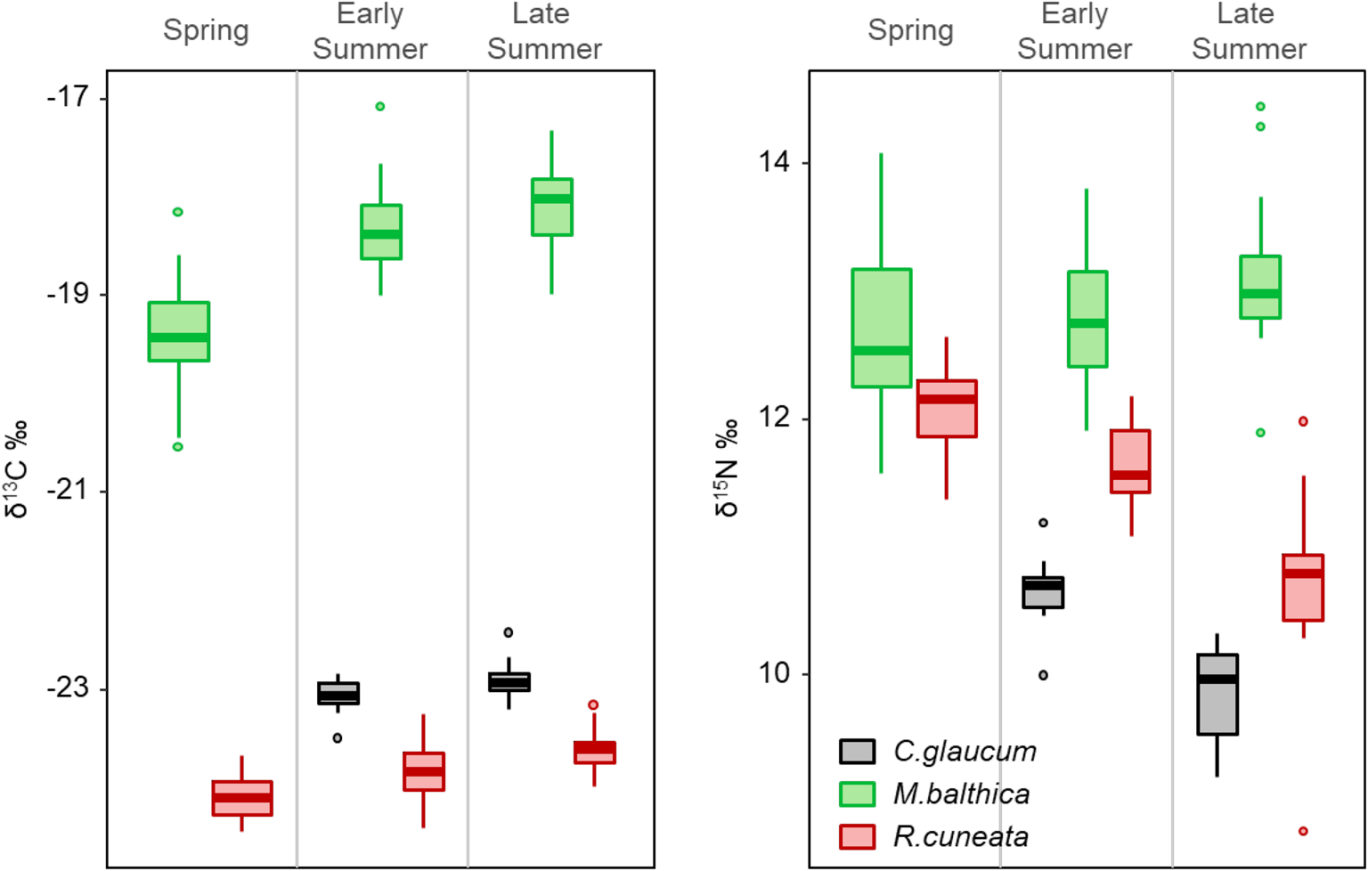
Seasonal change in δ^13^C and δ^15^N values for the native (*M. balthica, C. glaucum*) and non-native (*R. cuneata*) clams sampled at Brandalssund for the three timepoints (Spring, Early Summer and Late Summer). Values are median (lines), first and third quartiles (hinges), outliers (dots).

For all clams there was an increase to more enriched ^13^C values over the season (Fig. 4): *R. cuneata* δ^13^C values were significantly more ^13^C depleted in spring compared to summer time (KW, H_(2, 60) =_ 22.92, p < 0.001) as well as for *M. balthica* (KW, H_(2, 60)_ = 34.07, p < 0.001). For *C. glaucum* the significant change occurred between early and late summer (MW, Z_(1, 33)_ =  − 2.46, p = 0.014).

*R. cuneata* δ^15^N significantly decreased over the season (Fig. 3, KW, H_(2, 60)_ = 37.98, p < 0.001, all timepoints significantly different from each other in pairwise post hoc comparisons). *C. glaucum* also showed a decrease in δ^15^N values from early to late summer (MW, Z_(1, 40)_ = 4.64, p < 0.001). Finally, the δ^15^N value for *M. balthica* was unchanged over time or a slightly increased (Fig. 4, KW, H_(2, 60)_ = 5.56, p = 0.06).

### Species specific difference and temporal change in fatty acid composition

**Figure 5:**
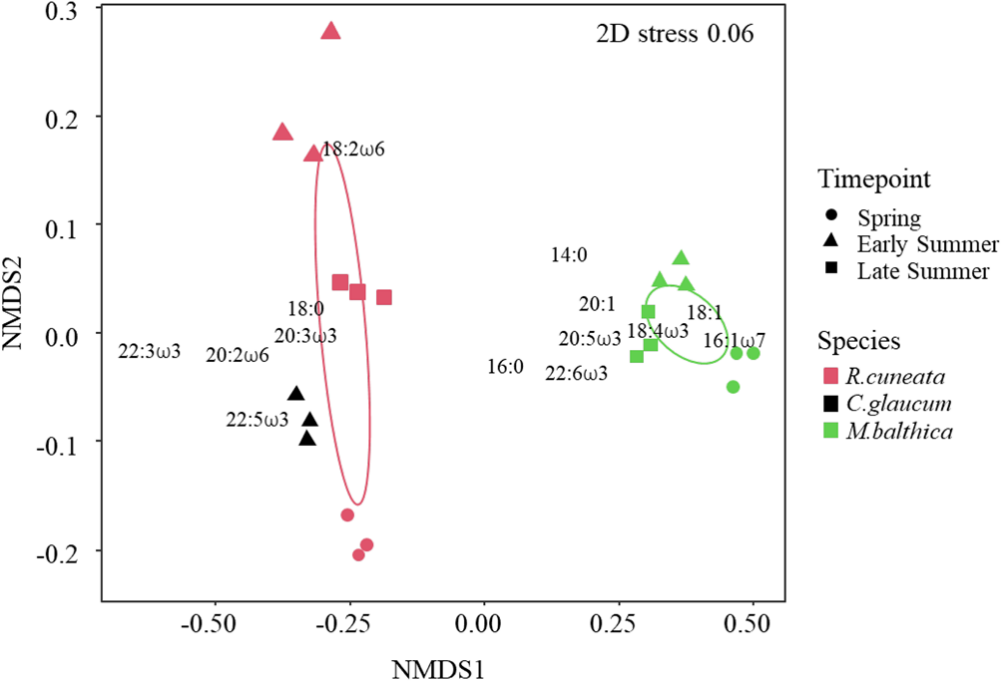
Non-metric multidimensional scaling (nMDS) based on Bray–Curtis dissimilarity matrix calculated on fatty acid (FA %) for the native (*M. balthica* (green)*, C. glaucum* (black)) and non-native (*R. cuneata* (red)) clams sampled at Brandalssund for the three timepoints (Spring = circles, Early Summer = triangles, Late Summer = squares). Each symbol is a composite sample of five individuals, see "Methods" for details. Ellipses (set to 40% of the data, i.e. core trophic niche) represent the FA niche. The 14 FAs shown are explaining up to 80% of the average dissimilarity between species (see text for details).

There was a clear separation among the three clams analyzed for fatty acid composition (Brandalssund site only) although *M. balthica* deviated the most (Fig. 5, Table 1). During early summer when data for the three species were available, *M. balthica* had the highest proportion of the essential fatty acids DHA (docosahexaenoic acid, 22:6ω3, KW, H_(2, 9)_ = 5.96, p = 0.05) and EPA (eicosapentaenoic acid, 20:5ω3, KW, H_(2, 9)_ = 7.20, p = 0.02) and of the diatom marker 16:1ω7 (KW, H_(2, 9)_ = 7.20, p = 0.02), while *C. R. cuneata* had highest proportion of the cyanobacterial markers 18:3ω3 and 18:2ω6 (KW, H_(2, 9)_ = 7.20, p = 0.03, in both cases, Table 1).

**Table 1:** Data (mean ± sd) of individual fatty acids and groups (% of total FA) and total FA (mg FA g^−1^) of the native and non-native clam species sampled at different timepoint. *Essential FAs (EFAs: 20:4ω6, 20:5ω3, 22:6ω3).

% of total FA	*C.glaucum*	*M. balthica*			*R. cuneata*		
	Early Summer	Spring	Early Summer	Later Summer	Spring	Early Summer	Later Summer
14:0	4.33 ± 0.39	5.05 ± 0.28	3.60 ± 0.19	2.27 ± 0.10	1.29 ± 0.11	5.04 ± 0.32	1.79 ± 0.10
14:1ω5	0,00 ± 0.00	0.09 ± 0.02	0.08 ± 0.02	0.04 ± 0.01	0.06 ± 0.01	0.01 ± 0.02	0.02 ± 0.02
15:0*i*	0.11 ± 0.02	0.07 ± 0.01	0.13 ± 0.02	0.10 ± 0.00	0.10 ± 0.00	0.10 ± 0.01	0.11 ± 0.02
15:0*ai*	0.10 ± 0.02	0.04 ± 0.01	0.00 ± 0.00	0.00 ± 0.0	0.05 ± 0.01	0.00 ± 0.00	0.02 ± 0.03
15:0	0.47 ± 0.01	0.29 ± 0.01	0.36 ± 0.02	0.51 ± 0.06	0.62 ± 0.03	0.39 ± 0.02	0.42 ± 0.01
16:0*i*	0.29 ± 0.03	0.13 ± 0.01	0.22 ± 0.02	0.27 ± 0.02	0.18 ± 0.01	0.03 ± 0.04	0.12 ± 0.00
16:0	11.76 ± 0.52	10.09 ± 0.11	10.01 ± 0.25	9.73 ± 0.23	11.72 ± 0.57	8.73 ± 6.07	13.59 ± 0.25
16:1ω7	4.24 ± 0.40	12.81 ± 0.37	12.52 ± 0.89	11.22 ± 1.14	4.56 ± 0.26	2.42 ± 0.10	6.23 ± 0.67
17:0*i*	1.056 ± 0.09	0.24 ± 0.04	0.51 ± 0.03	0.63 ± 0.06	1.54 ± 0.11	0.82 ± 0.08	0.85 ± 0.05
17:0*ai*	0.92 ± 0.05	0.11 ± 0.02	0.24 ± 0.01	0.35 ± 0.08	0.76 ± 0.04	0.49 ± 0.05	0.66 ± 0.06
17:0	0.77 ± 0.09	0.11 ± 0.01	0.20 ± 0.01	0.27 ± 0.02	0.92 ± 0.02	0.57 ± 0.05	0.70 ± 0.06
18:0*i*	0.00 ± 0.00	0.17 ± 0.01	0.00 ± 0.00	0.00 ± 0.00	0.47 ± 0.04	0.05 ± 0.06	0.00 ± 0.00
16:4ω3	0.00 ± 0.00	0.10 ± 0.00	0.00 ± 0.00	0.00 ± 0.00	0.00 ± 0.00	0.05 ± 0.08	0.00 ± 0.00
18:0	4.94 ± 0.21	1.49 ± 0.14	1.74 ± 0.21	1.97 ± 0.04	5.60 ± 0.20	6.64 ± 0.27	5.93 ± 0.34
18:1	3.44 ± 0.37	13.14 ± 0.26	12.04 ± 0.88	13.66 ± 0.42	5.10 ± 0.17	3.67 ± 0.05	4.48 ± 0.32
18:1ω7	2.56 ± 0.45	1.16 ± 0.20	1.25 ± 0.02	1.27 ± 0.07	2.72 ± 0.11	1.55 ± 0.18	3.55 ± 0.08
18:2ω6	4.44 ± 0.57	1.07 ± 0.09	2.04 ± 0.19	1.90 ± 0.14	1.38 ± 0.05	7.03 ± 0.65	2.21 ± 0.14
18:3ω6	0.00 ± 0.00	0.21 ± 0.01	0.21 ± 0.01	0.22 ± 0.02	0.00 ± 0.00	0.18 ± 0.01	0.24 ± 0.01
19:0	0.00 ± 0.00	0.00 ± 0.00	0.00 ± 0.00	0.00 ± 0.00	0.0 ± 0.00	0.26 ± 0.02	0.21 ± 0.03
18:3ω3	1.86 ± 0.21	0.71 ± 0.07	1.16 ± 0.04	1.02 ± 0.09	2.39 ± 0.30	2.51 ± 0.20	2.74 ± 0.27
18:4ω3	1.71 ± 0.18	6.62 ± 0.31	4.47 ± 0.28	3.21 ± 0.16	3.35 ± 0.22	3.34 ± 0.21	3.21 ± 0.16
20:0	0.00 ± 0.00	0.00 ± 0.00	0.00 ± 0.00	0.00 ± 0.00	0.13 ± 0.18	0.39 ± 0.03	0.34 ± 0.04
20:1	2.91 ± 0.31	6.48 ± 0.30	6.28 ± 0.48	7.17 ± 0.45	3.59 ± 0.26	3.94 ± 0.29	4.77 ± 0.18
20:1ω9	2.64 ± 0.01	1.43 ± 0.06	1.22 ± 0.14	1.38 ± 0.14	2.15 ± 0.20	1.12 ± 0.12	1.56 ± 0.02
20:2ω6	7.48 ± 0.51	0.61 ± 0.09	0.96 ± 0.14	0.96 ± 0.12	2.37 ± 0.07	4.30 ± 0.14	2.80 ± 0.21
20:3ω6	0.41 ± 0.03	0.00 ± 0.00	0.00 ± 0.00	0.00 ± 0.00	0.28 ± 0.03	0.32 ± 0.06	0.38 ± 0.03
21:0	0.00 ± 0.00	0.00 ± 0.00	0.00 ± 0.00	0.00 ± 0.00	0.00 ± 0.00	0.12 ± 0.17	0.00 ± 0.00
20:3ω3	6.95 ± 0.93	0.93 ± 0.10	1.52 ± 0.05	1.91 ± 0.10	2.91 ± 0.03	4.74 ± 0.18	2.89 ± 0.17
20:4ω6*	0.72 ± 0.07	0.21 ± 0.03	0.33 ± 0.02	0.37 ± 0.01	0.73 ± 0.14	0.58 ± 0.06	0.69 ± 0.06
20:5ω3*	8.50 ± 0.27	10.49 ± 0.52	12.07 ± 0.55	10.86 ± 0.22	9.00 ± 0.59	6.76 ± 0.34	11.16 ± 0.17
22:3ω3	2.24 ± 0.33	0.00 ± 0.00	0.00 ± 0.00	0.00 ± 0.00	2.46 ± 0.34	2.62 ± 0.25	2.10 ± 0.19
22:5ω3	3.20 ± 0.42	0.96 ± 0.15	1.33 ± 0.11	1.55 ± 0.11	9.59 ± 0.51	5.69 ± 0.52	8.11 ± 0.30
22:6ω3*	21.98 ± 1.01	25.19 ± 0.65	25.53 ± 0.96	27.15 ± 0.69	23.59 ± 1.19	21.11 ± 1.40	18.11 ± 0.68
FA groups (% of total)							
Σ SFA	22.27 ± 3.9	17.0 ± 3.4	15.9 ± 3.3	14.8 ± 3.1	20.3 ± 3.9	22.1 ± 3.3	23.0 ± 4.4
Σ MUFA	15.8 ± 1.3	35.1 ± 5.4	33.4 ± 5.1	34.7 ± 5.3	18.2 ± 1.7	12.7 ± 1.4	20.6 ± 2.1
Σ PUFA	59.5 ± 5.7	47.1 ± 6.9	49.6 ± 7.0	49.2 ± 7.3	58.4 ± 6.3	59.2 ± 5.3	54.7 ± 5.1
Σ BrFA	2.5 ± 0.4	0.7 ± 0.1	1.1 ± 0.2	1.4 ± 0.2	3.1 ± 0.5	1.5 ± 0.3	1.8 ± 0.3
Σ EFA*	31.2 ± 8.8	35.9 ± 10.3	37.9 ± 10.3	38.4 ± 11.0	33.7 ± 9.6	28.4 ± 8.6	30.0 ± 7.2
Σ ω3	46.4 ± 6.7	45.0 ± 8.2	46.1 ± 8.3	45.7 ± 8.7	53.7 ± 7.2	46.8 ± 6.1	48.3 ± 5.7
Σ ω6	13.0 ± 2.9	2.1 ± 0.4	3.5 ± 0.7	3.5 ± 0.7	4.8 ± 0.8	12.4 ± 2.7	6.3 ± 2.0
total FA (mg FA g^−1^)	99 ± 19	217 ± 33	281 ± 40	177 ± 25	142 ± 24	132 ± 35	82 ± 16

*R. cuneata* had a large difference in fatty acid composition among the different timepoints while *M. balthica* had a more uniform fatty acid composition over time (Fig. 5, Table 1). There was an effect of timepoint (PERMANOVA_(2)_, p < 0.0001) and species (PERMANOVA_(2)_, p < 0.0001) on the fatty acid composition as well as an interaction effect between time and the two species (PERMANOVA_(2)_, p < 0.0001). The 14 individual fatty acids that were responsible for 80% of the variation in the fatty acid profiles were: 14:0, 16:0, 16:1ω7, 18:0, 18:1, 18:2ω6, 18:4ω3, 20:1, 20:2ω6, 20:3ω3, 20:5ω3 (EPA), 22:3ω3, 22:5ω3 and 22:6ω3 (DHA).

Regarding absolute content of the different groups of FAs (Table 1), there was no change in polyunsaturated fatty acid (PUFA) over time for *R. cuneata* (KW, H_(2, 9)_ = 2.80, p = 0.25), neither for monounsaturated fatty acid (MUFA) content (KW, H_(2, 9)_ = 5.60, p = 0.06) or saturated fatty acid (SFA) content (KW, H_(2, 9)_ = 1.69, p = 0.43). There was also no significant change for *M. balthica* between timepoints in PUFA content (KW, H_(2, 9)_ = 1.92, p = 0.38), MUFA content (KW, H_(2, 9)_ = 4.36, p = 0.11) but a decrease in SFA content over time (KW, H_(2, 9)_ = 5.96, p = 0.05).

## Discussion

This study shows trophic niche separation between the non-indigenous clam *Rangia cuneata* and the Baltic Sea native clams, the suspension feeders *Cerastoderma glaucum*, *Mya arenaria* and the facultative suspension deposit feeder *Macoma balthica*. The low niche overlap, as assessed from both stable isotope and fatty acid niche analyses, supports the existence of a vacant trophic niche in the species poor shallow sediment habitats of the Baltic. The mechanism explaining niche separation among these clams could be rather subtle resource partitioning, for example through different selectivity in ingestion or absorption efficiency regarding size or species of phytoplankton. Below we discuss the isotope and fatty acid results in detail and the consequences of our results for the Baltic Sea ecosystem and management in the context of eutrophication and biodiversity conservation.

Generally, *R. cuneata* had the most depleted ^13^C values of the clams, regardless of site or timepoint sampled. Such depleted values indicate high reliance of pelagic carbon sources, i.e. phytoplankton^36^. An alternative explanation to low δ^13^C values which confound its use as a pelagic biomarker could be a lower trophic discrimination factor for ^13^C (or ^15^N) for this species compared to the other clams (Martinez del Rio et al., 2005). Still, *R. cuneata* showed pronounced seasonal changes in both the nitrogen isotope and fatty acid composition, likely reflecting the seasonal changes occurring in the phytoplankton community, indicative of a generalist feeding behavior, i.e. trophic plasticity. During the summer bloom of nitrogen fixing cyanobacteria, the δ^15^N of planktonic material becomes more depleted^37^. Accordingly, δ^15^N for *R. cuneata* (and *C. glaucum*) decreased throughout summer indicating that nitrogen-fixing cyanobacteria are an important ultimate nitrogen source for these suspension-feeders. Fatty acid composition indicates a higher content of the cyanobacteria biomarkers 18:2ω6 and 18:3ω3 for *R. cuneata* compared to the other species. These biomarkers are largely present in the bloom-forming cyanobacteria species during summer in the Baltic Proper^35,38^. Utilizing cyanobacteria as a food source is not unique to *R. cuneata* though, the native suspension-feeding blue mussel, *Mytilus edulis trossulus*, that dominate hard bottoms (but does not exist in these sedimentary habitats) is known to feed actively on N-fixing filamentous cyanobacteria^35^.

Overall, a high proportion of the essential fatty acids 22:6ω3 and 20:5ω3 and a generally high ω − 3 content indicate dietary reliance of diatoms and dinoflagellates, commonly considered high quality food sources for benthos in temperate areas^38^. Our recent experimental work on Baltic blue mussels also supports these fatty acids as diatom biomarkers in the Baltic Sea^35^. *M. balthica* appears to have a more diatom-based diet compared to *R. cuneata* as it shows higher 16:1ω7 values as well as 20:5ω3 (DHA) values^39,40^. The more pronounced seasonal change in fatty acid composition of *R. cuneata* suggests a greater trophic plasticity of this species as previously mentioned, compared to *M. balthica* which seems more selective in what is assimilated relative to what is available in the planktonic material over time. Still, the isotope niche size was, as expected from its facultative suspension-deposit feeding mode^41^, broadest for *M. balthica* which has the ability to feed both on aged organic material in the sediment and fresh planktonic material. Overall, the isotopic niche separation among the clams supports the diet separation seen in fatty acid data; *M. balthica* rely on diatoms mostly present during spring bloom and *R. cuneata* seems to preferentially utilize the cyanobacterial summer bloom. This might be a result of the cold-adapted *M. balthica* having its main growth period during spring^42^ while the sub-tropical *R. cuneata* has its main growth period at higher temperatures in late summer^30^.

In north America, where *R. cuneata* has experienced a large increase in population density and range along the Atlantic coast during the last 40 years^43^, experimental studies have shown that *M. balthica* switch to deposit feeding when *R. cuneata* biomass is high^44^. The higher δ^15^N values, which is generally an indication of feeding on aged organic matter such as sediment particles in deposit feeders^10^ or by feeding on benthic diatoms^45^, and the more uniform isotope and fatty acid composition over time in *M. balthica* observed in our study supports a predominantly deposit feeding mode and/or a more selective feeding of specific phytoplankton (diatoms) and may indicate that a similar shift is taking place in the Baltic Sea. On the other hand, isotope studies from shallow habitats in the Baltic where R. *cuneata* has not yet been found have shown that *M. balthica* also have consistently higher δ^15^N and more enriched ^13^C values than the two suspension feeders, *M. arenaria* and *C. glaucum* in areas where the three species co-occur^46^. This suggests resource partitioning among the native clam species where the high δ^13^C and δ^15^N values of *M. balthica* support the assimilation of benthic organic matter. Experimental field studies testing the effect of *R. cuneata* presence on *M. balthica* in the Baltic would confirm if *M. balthica* has indeed changed its feeding pattern after *R. cuneata* established its population. Potential changes in trophic behavior and resource partitioning may have long-term consequences on food web interactions and species distribution patterns as well as on the recycling of biogeochemical elements. For example, the addition of a non-indigenous polychaete species has at the same time lead to a more complete utilization of resources from a secondary production perspective^9,10^ and a net release of bioavailable nutrients from its bioturbation activities which could counteract eutrophication mitigation^47,48^.

The Baltic Sea is evolutionary young with the present salinity conditions existing only for some 3000 years (Snoeijs-Leijonmalm et al., 2017). Only relatively few species have adapted to this existing brackish environment, but new species are regularly added by natural immigration or man-made introductions^23,24^. Only few bivalves are widely distributed and of significance, i.e. *Mytilus edulis trossulus* on hard substrates, and *M. balthica, C. glaucum* and *M. arenaria* on sediment bottoms. In fact, one of these, *M. arenaria*, is also an immigrant to the Baltic Sea probably transported with Viking ships from North America during mediaeval time c.1000-1350 AD^49^. Little competition with other species has resulted in *M. balthica, C. glaucum and M. arenaria* expanding their niches and distribution in the Baltic Sea with *M. balthica* dominating the overall biomass on soft sediments and is found from 0 to 90 m depth^50,51^. Even if the present expansion of *R. cuneata* is very rapid in the Baltic Sea, it is likely that it will mainly colonize shallow areas and bays with somewhat reduced salinity and not directly affect the open Baltic.

**Figure 6:**
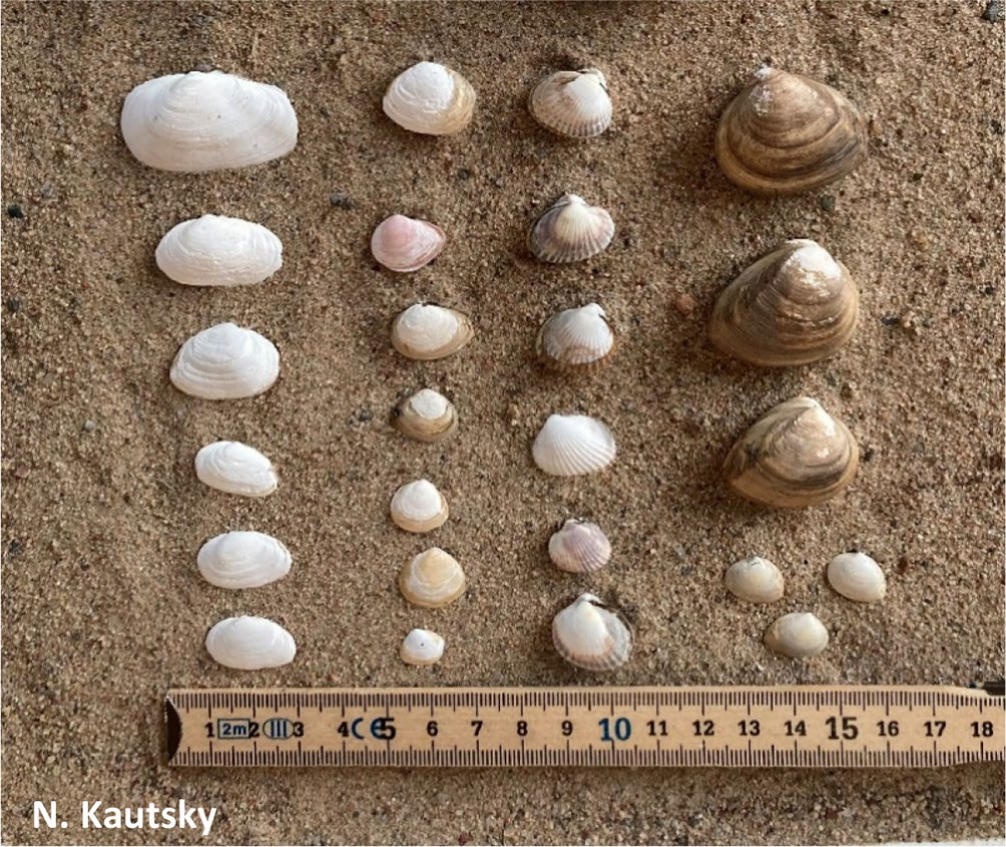
Photo showing typical size distribution of the four bivalves found at Brandalssund, from left to right: *Mya arenaria*, *Macoma balthica*, *Cerastoderma glaucum* and *Rangia cuneata*. The larger shells of *R. cuneata* found at this sampling site were 4–5 years old and the smallest *R. cuneata* belong to the 1+ year class. Credits N. Kautsky.

While bivalves are generally smaller in the Baltic Sea than in marine areas, the large size of *R. cuneata* shows that it seems well adapted to brackish conditions (Fig. 6). However, temperature in the boreal Baltic is much lower than in North America^28^ which might affect *R. cuneata* reproduction^29^. The maximum age found for *R. cuneata* in Brandalssund and Tulka Byväg was 7 years, and in Sveden 6 years, indicating that these clams were recruited in 2014 or 2015 from parent populations that colonized this northernmost reported location a few years earlier (Fig. S1). The lack of complete year classes and the only very low number of young individuals (age 0–2 years) found at Brandalssund and Tulka Byväg suggests that recruitment success varies between years. The significant decrease in body condition for *M. balthica*, but not for *R. cuneata*, in late summer compared to early summer suggests that spawning occurred in between the two timepoints for the native clam (Fig. S2). In contrast, the absence of a decrease in body condition for *R. cuneata* suggests that they have not yet spawned at the later timepoint. They might spawn later in the year or not at all during the studied year (2021). Such irregular spawning might be expected to occur close the northern distribution limit for *R. cuneata.*

Even though we found that *R. cuneata* may occupy a vacant niche, we cannot exclude the possibility that the addition of biomass from *R. cuneata* will affect the total phytoplankton availability or the composition of it (e.g. preferential utilization of cyanobacteria). However, at the site Sveden, no other native clams were found at all, highlighting that *R. cuneata* constitutes an addition of an entire new functional group in certain areas, possibly improving water clarity and contributing to eutrophication mitigation^28^. Experimental studies manipulating the presence and absence of *R. cuneata* in natural communities would reveal if the native species (not only clams) shift their niche as a response to the *R. cuneata* addition and potential consequences for biogeochemical cycling and ecosystem functions. To conclude, the difference in both stable isotope niche and fatty acid profiles between the new species *R. cuneata* and the native bivalves strongly indicates trophic niche separation and supports the idea of vacant niches rather than competition for food in this species poor and eutrophicated system.

### Supplementary Information

Supplementary Information.

## Data Availability

The datasets used and/or analyzed during the current study available from the corresponding author on reasonable request.
