# Supplementary material for: Resource partitioning of a Mexican clam in species-poor Baltic Sea sediments indicates the existence of a vacant trophic niche
Source: Sci Rep. 2024 May 31;14:12527. doi: 10.1038/s41598-024-62832-3 (PMC11143366; doi:10.1038/s41598-024-62832-3)
Supplement: Supplementary file 1 — Supplementary Information. [file 41598_2024_62832_MOESM1_ESM.pdf]

# Resource partitioning of a Mexican clam in the species-poor Baltic Sea indicate the existence of a vacant trophic niche

Agnes ML Karlson[1,2], Nils Kautsky[1], Matilda Granberg[1], Andrius Garbaras[3], Hwanmi Lim[4], Camilla Liénart*[1,5]

[1] Department of Ecology, Environment and Plant sciences, Stockholm University, Stockholm, Sweden
[2] Stockholm University Baltic Sea Centre, Stockholm, Sweden
[3] Center for Physical Sciences and Technology, Vilnius, Lithuania
[4] Lipidor AB, Svärdvägen 13, SE-182 33 Danderyd, Sweden
[5] Université de Bordeaux, CNRS, Bordeaux INP, EPOC, UMR 5805, 33120 Arcachon, France
Corresponding author*: camilla.lienart@su.se

## Supplementary information

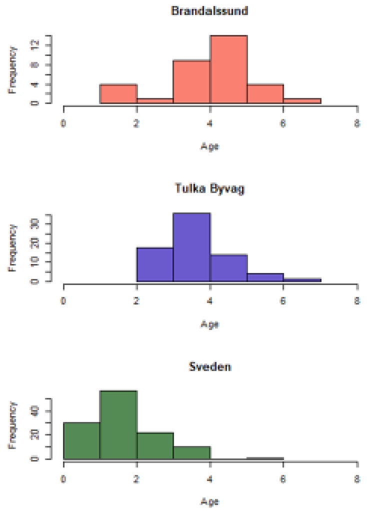


**Fig. S1**: Histogram of the age distribution of *R. Cuneata* (counts) in late summer in the three studied locations.

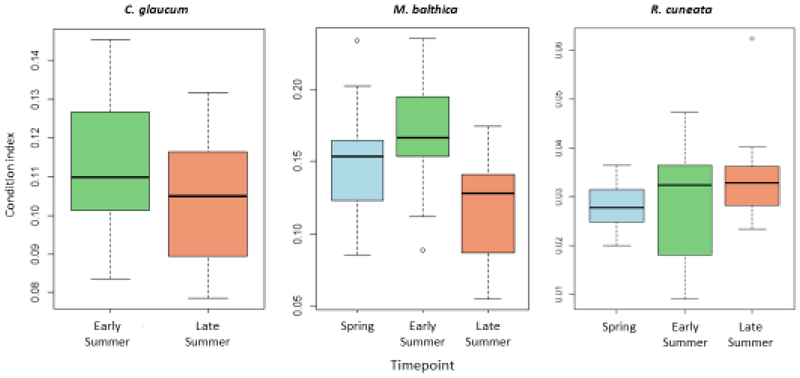


**Fig. S2**: Boxplot of condition index (soft tissue:shell, dry weight) of native and non-native clams collected at Brandalssund: *C. glaucum* for two timepoints, *M. balthica* and *R. cuneata* for three timepoints.
